# Supplementary material for: Direct measurement of engineered cancer mutations and their transcriptional phenotypes in single cells
Source: Nat Biotechnol. 2023 Sep 11;42(8):1254–62. doi: 10.1038/s41587-023-01949-8 (PMC11324510; doi:10.1038/s41587-023-01949-8)

nature biotechnology

Article

https://doi.org/10.1038/s41587-023-01949-8

Direct measurement of engineered cancer mutations and their transcriptional phenotypes in single cells

In the format provided by the authors and unedited

Supplementary Information

Table of contents.

Supplementary Figure 1. Dot plot showing the proportion of each genetic variant detected from single-cell cDNA and genomic DNA from *RACK1* edited HEK293T cells.

Supplementary Figure 2. Statistics of sgRNA libraries targeting *TP53* genetic variants.

Supplementary Figure 3. Multiple genetic variants can be introduced by one sgRNA.

Supplementary Figure 4. Dot plot showing the proportion of each genetic variant detected from single-cell cDNA and genomic DNA.

Supplementary Figure 5. UMAP plot of HCT116 cells which various *TP53* genetic variants are introduced by full sgRNA library.

Supplementary Figure 6. Violin plot showing P53 pathway gene expression score per cells with synonymous mutations in HCT116 cells.

Supplementary Figure 7. UMAP plot of U2OS cells which various *TP53* genetic variants are introduced by full sgRNA library.

Supplementary Figure 8. Numbers of *TP53* mutations analyzed in each cell-line.

Supplementary Figure 1. Dot plot showing the proportion of each genetic variant detected from single-cell cDNA and genomic DNA from *RACK1* edited HEK293T cells.

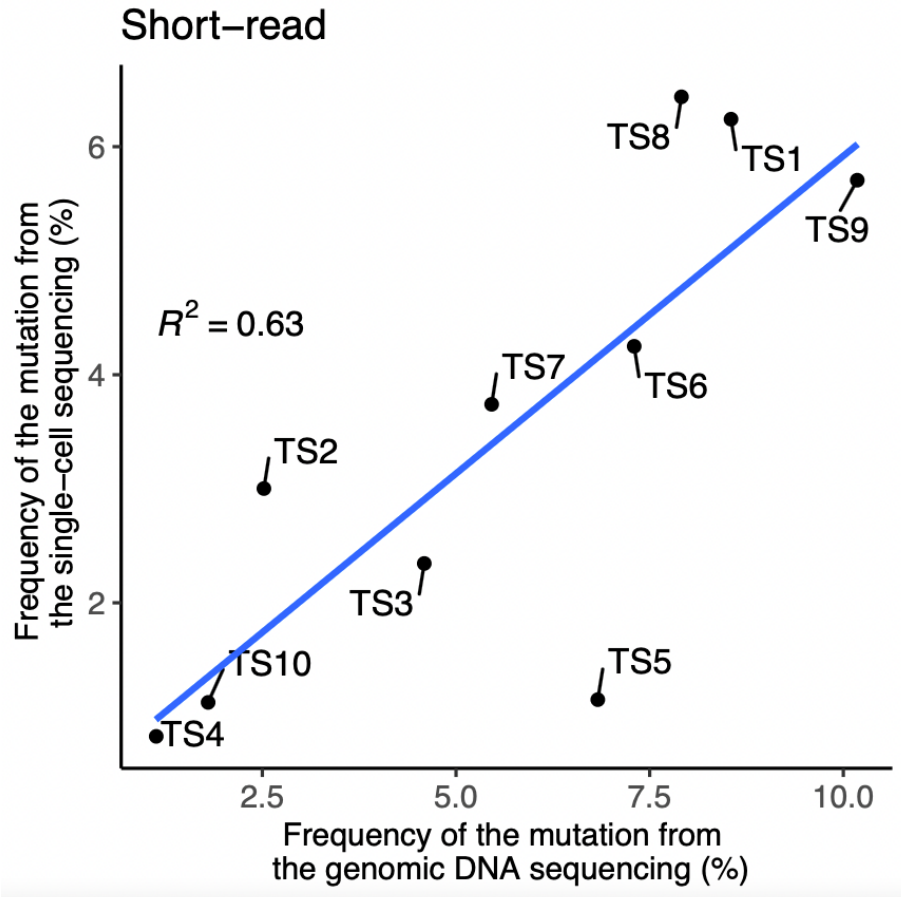

Supplementary Figure 2. Statistics of sgRNA libraries targeting *TP53* genetic variants.

	sgRNA	Targetable variants	Possible variants
NGG_Base_Editors	74	99/351(28.21%)	920
NG_Base_Editors	88	159/351(45.30%)	1999
Total	162	251/351(71.51%)	2892

Supplementary Figure 3. Multiple genetic variants can be introduced by one sgRNA. Underlines indicate each triplet codon and number indicate position of the codon. Red DNA sequences indicate substituted bases and blues indicate PAM sequences.

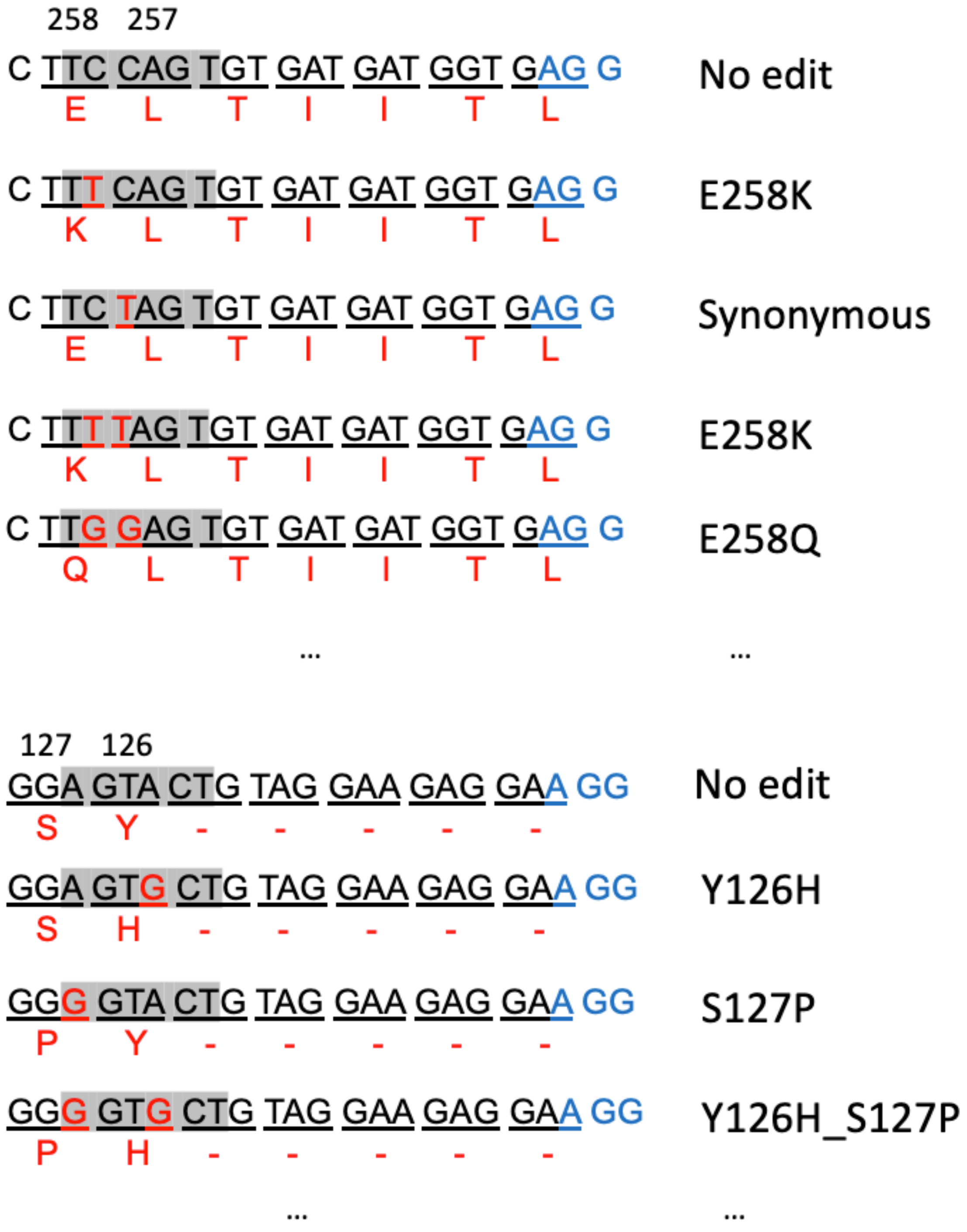

Supplementary Figure 4. Dot plot showing the proportion of each genetic variant detected from single-cell cDNA and genomic DNA. Genetic variants generating premature stop codon are removed.

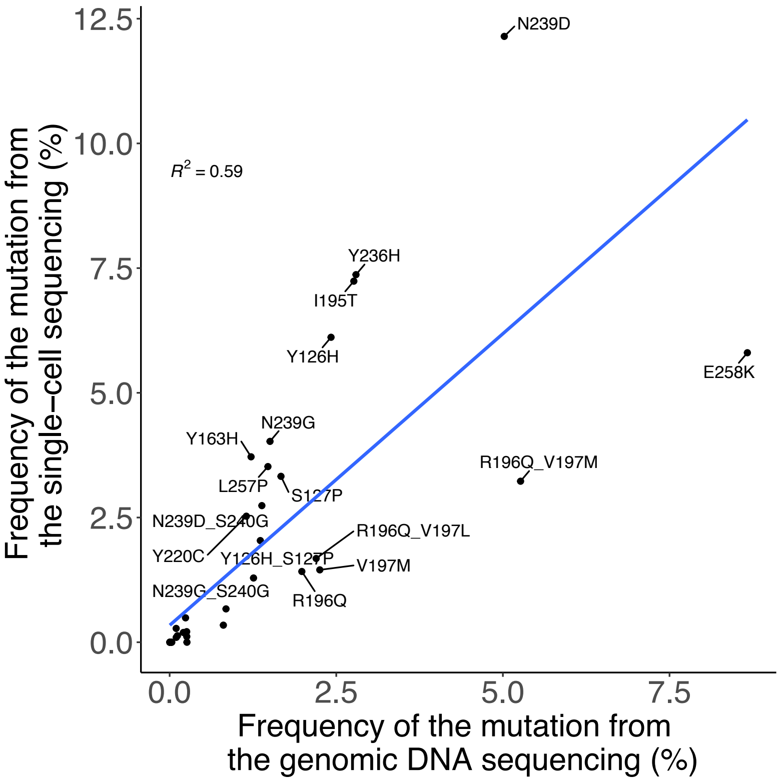

Supplementary Figure 5. UMAP plot of HCT116 cells which various *TP53* genetic variants are introduced by full sgRNA library.

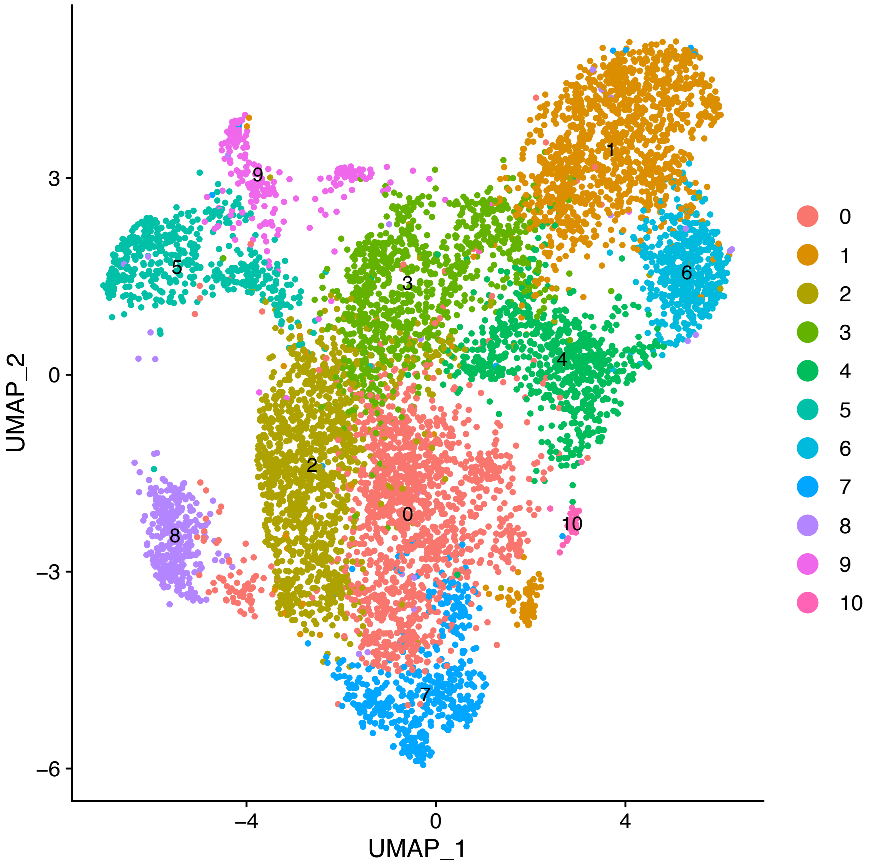

Supplementary Figure 6. Violin plot showing P53 pathway gene expression score per cells with synonymous mutations in HCT116 cells. N= 63, 5, 886 biologically independent cells for R280R, E287E and WT. Upper hinge: 75% quantile, middle line: 50% quantile, lower hinge: 25% quantile. Whisker means data range excluding outliers.

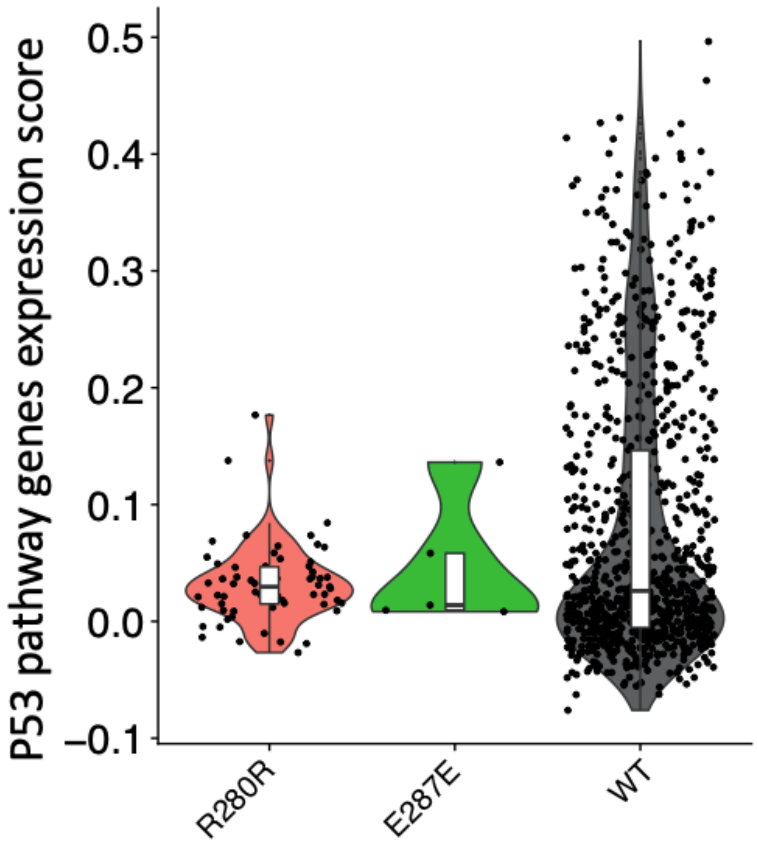

Supplementary Figure 7. UMAP plot of U2OS cells which various *TP53* genetic variants are introduced by full sgRNA library.

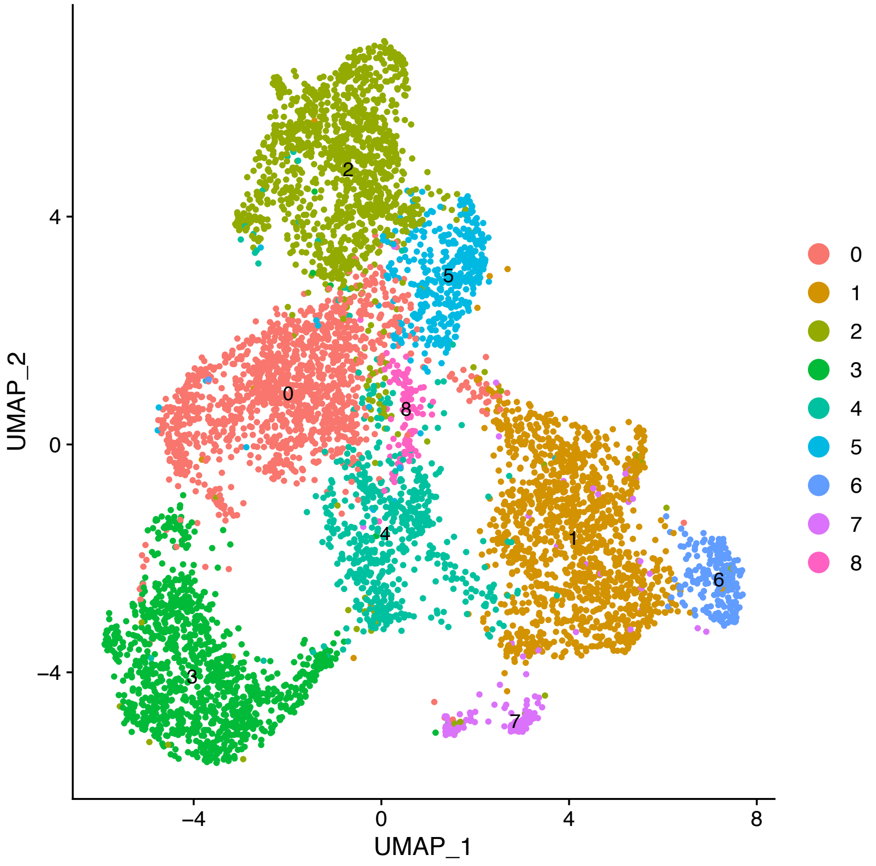

Supplementary Figure 8. Numbers of *TP53* mutations analyzed in each cell-line. The efficiency of CRISPR base editor can vary depending on the cell line. Therefore, an sgRNA that is effective in one cell line may not be as effective in another, resulting in preferential introduction of mutations. In addition, we excluded *TP53* mutations with less than 5 cells for the analysis.

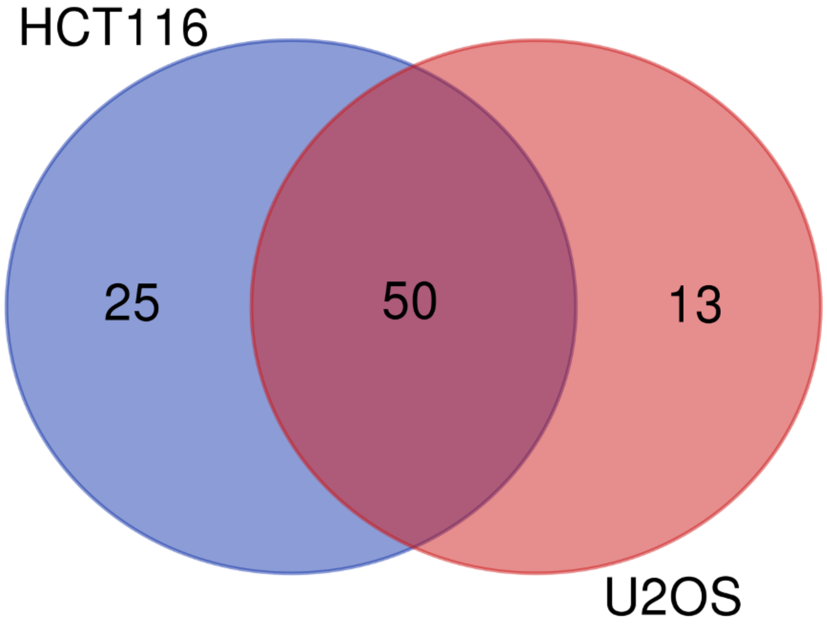

Supplement: Supplementary file 1 — Supplementary Figs. 1–8. [file 41587_2023_1949_MOESM1_ESM.pdf]
